# Adipose-Derived Stem Cells: Current Applications and Future Directions in the Regeneration of Multiple Tissues

**DOI:** 10.1155/2020/8810813

**Published:** 2020-12-10

**Authors:** Jiaxin Zhang, Yuzhe Liu, Yutong Chen, Lei Yuan, He Liu, Jincheng Wang, Qiran Liu, Yan Zhang

**Affiliations:** ^1^Department of Breast Surgery, The Second Hospital of Jilin University, Changchun 130041, China; ^2^Orthopaedic Medical Center, The Second Hospital of Jilin University, Changchun 130041, China; ^3^Department of Cardiovascular Surgery, The Second Hospital of Jilin University, Changchun 130041, China

## Abstract

Adipose-derived stem cells (ADSCs) can maintain self-renewal and enhanced multidifferentiation potential through the release of a variety of paracrine factors and extracellular vesicles, allowing them to repair damaged organs and tissues. Consequently, considerable attention has increasingly been paid to their application in tissue engineering and organ regeneration. Here, we provide a comprehensive overview of the current status of ADSC preparation, including harvesting, isolation, and identification. The advances in preclinical and clinical evidence-based ADSC therapy for bone, cartilage, myocardium, liver, and nervous system regeneration as well as skin wound healing are also summarized. Notably, the perspectives, potential challenges, and future directions for ADSC-related researches are discussed. We hope that this review can provide comprehensive and standardized guidelines for the safe and effective application of ADSCs to achieve predictable and desired therapeutic effects.

## 1. Introduction

Organ or tissue transplantation is a preferred treatment option for patients with terminal organ or tissue failure. In a retrospective study of data for a 25-year period using the United Network for Organ Sharing database, organ transplantation was associated with a significant survival benefit, saving over 2,270,859 life-years [1]. However, the World Health Organization estimates that only 10% of the global need for organ and tissue transplantation can be satisfied [2]. Inadequate tissue and organ supply remains a major public health challenge. Stem cells are particularly useful in the area of organ and tissue reconstruction, as they are abundant, can be harvested through minimally invasive methods, can be induced to differentiate into multiple cell lineages, and can be manufactured based on good manufacturing practice guidelines [3]. The application of stem cells has emerged as a means of compensating for the lack of tissue or organ availability and has resulted in a quantum leap in regenerative medicine [4].

Stem cell candidates include embryonic stem cells (ESCs), induced pluripotent stem cells (iPSCs), and adult stem cells, such as mesenchymal stromal cells (MSCs) [5]. The use of ESCs creates ethical concerns and can also evoke immune responses. Besides, iPSCs avoid ethical concerns and immune responses, but the cell preparation method is relatively complex and time-consuming [6]. Although MSCs can be found in diverse tissues, bone marrow-derived mesenchymal stem cells (BM-MSCs) and adipose-derived stem cells (ADSCs) have been the subject of more comprehensive, in-depth research [7]. ADSCs have several advantages over BM-MSCs. On the one hand, higher yields of ADSCs can easily be obtained from subcutaneous regions through a minimally invasive and painless procedure; furthermore, ADSCs can maintain their phenotype longer in culture, present a greater proliferative capacity [8], and may also be more suitable for allogenic transplantation than BM-MSCs [9]. Besides, ADSCs can differentiate into cell types of the three developmental germ layers (endoderm, mesoderm, and ectoderm), including adipocytes, osteoblasts, chondrocytes, neurocytes, and hepatocyte [10, 11]. These advantages render ADSCs to be the most attractive source of MSCs for regenerative medicine. Meanwhile, currently, the clinical approaches involving ADSCs gradually increased.

For the effective use of ADSCs in the regeneration of different types of tissue, the recent research progress regarding the use of these stem cells in the field of tissue engineering must be evaluated. Above all, the different ADSC harvesting methods can affect the basic properties of the cells, such as their ability to proliferate and their antiapoptotic capacity [12]. The gene expression patterns and the tendency toward specific germ layer differentiation will also be affected by the harvesting method [13]. Furthermore, because monolayers cannot mimic the interactions between cells and the extracellular matrix (ECM), ADSCs expanded as a 2-dimensional (2D) monolayer lose their ability to proliferate, differentiate, and form colonies after several passages [14]. Compared with the 2D environment, 3D culture enhances ADSC osteogenic differentiation, increases matrix mineralization, and enhances ADSC viability during proliferation [15].

A main point of concern in the field of tissue engineering is the maintenance of homeostasis in the ADSC microenvironment. A 3D scaffold architecture typically comprises porous, biocompatible, and biodegradable materials that provide a suitable microenvironment for stimulating cell growth and function [16]. The porosity and pore size of scaffolds can have direct effects on their function, including the mass transport of oxygen and vital nutrients necessary for stem cell proliferation, differentiation, and migration [17, 18]. In addition to the factors mentioned above, various cytokines can also enhance the proliferative and migratory ability of ADSCs, as well as promote their differentiation [19]. Under an ischemic environment, cytokines play a vital role in ADSC-mediated promotion of the recovery of blood supply and wound healing through the induction of angiogenesis [20]. In an inflammatory environment, those additional effects of cytokines can enhance the secretion of angiogenic and anti-inflammatory factors by ADSCs [21].

**Scheme 1:**
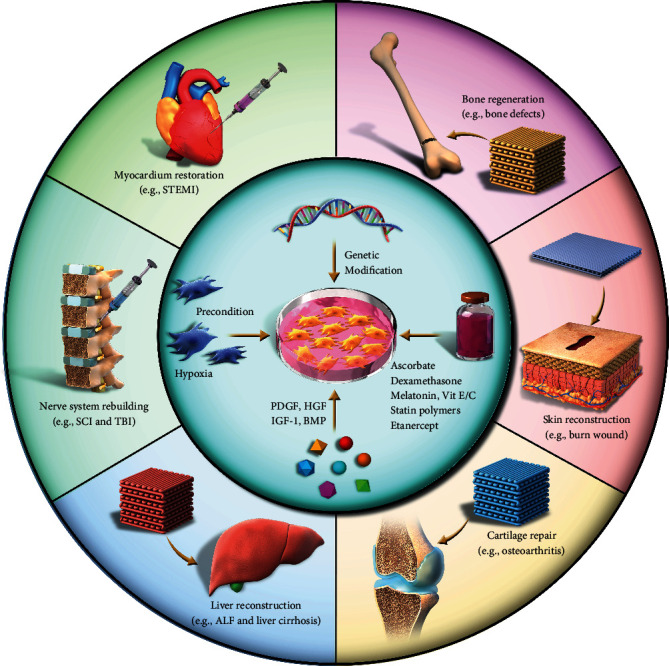
Schematic representation of the applications for ADSC-based therapies in regenerative medicine. PDGF: platelet-derived growth factor; HGF: hepatocyte growth factor; IGF-1: insulin-like growth factor 1; BMP: bone morphogenetic protein; SCI: spinal cord injury; TBI: traumatic brain injury; ALF: acute liver failure; STEMI: ST-elevation acute myocardial infarction.

Recent reviews in related areas have not highlighted or detailed the optimal methods for the preparation of ADSCs, the latest progress in the application of ADSCs in various organs, or the potential risk for tumor invasiveness associated with ADSC-cancer cell interactions [22]. The following sections shed light on the methods for harvesting, isolating, preserving, and identifying ADSCs. Furthermore, we discuss the novel clinical uses for ADSCs as regenerative therapies, including bone regeneration, cartilage repair, nerve system rebuilding, liver regeneration, myocardium restoration, and skin regeneration (Scheme 1). The current challenges for the use of ADSCs in the field of regenerative medicine are summarized to provide directions for their clinical application.

## 2. Preparation of ADSCs

**Figure 1:**
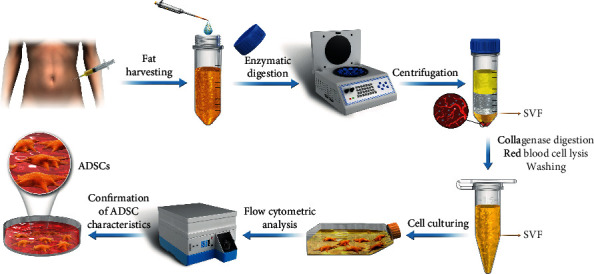
The typical process for the preparation of ADSCs from human adipose tissue. SVF: stromal vascular fraction.

A rich source of ADSCs is an essential foundation for the extensive investigation and application of regenerative medicine. ADSC applications sourced these cells from subcutaneous adipose tissues obtained by aspiration, liposuction, or excision. Then, the most widely utilized approach to isolate ADSCs from the obtained fragments relies on collagenase digestion, followed by centrifugation. Finally, the isolated ADSCs are proliferated in conventional culturing conditions and distinguished from other cells by flow cytometry (Figure 1).

### 2.1. Harvesting of ADSCs

Current methods for harvesting ADSCs include aspiration, liposuction, and direct excision. Coleman's aspiration technique is currently the most commonly used method for the collection of adipose tissue, which relies on the slight negative pressure with a syringe. Furthermore, the negative pressure (<250 mmHg) of liposuction-related methods by motor could harvest a large volumetric adipose tissue. Liposuction-related methods include conventional, ultrasound-assisted liposuction, power-assisted liposuction, and laser-assisted. And direct excision could harvest a piece of adipose tissue, and the obtained fragments require mincing into tiny particles with the use of surgical blades [23].

The yield and properties of ADSCs may differ according to multiple variables, such as the harvesting method, the adipose tissue depot, medical comorbidities of the patient, body mass index (BMI), and age. There is evidence that harvesting adipose tissue by aspiration halves the concentration of ADSCs compared with harvesting by excision [24]. The yield and biological characteristics of viable ADSCs obtained by excision are significantly improved when compared with those obtained through liposuction [25]. The gene expression pattern and the tendency toward differentiation into a specific germ layer can also be affected by the harvesting method. ADSCs collected through direct excision tend toward mesodermal and ectodermal differentiation, whereas those obtained by liposuction are more likely to differentiate into endoderm [13].

The collection location also affects the yield and differentiation capability of ADSCs. There is some evidence showing that the thigh provides a better yield of ADSCs than the abdomen, waist, and inner knee [26]. In contrast, there is no significant difference in cell viability among the donor areas. ADSC yields and differentiation potential are also reported to be higher in subcutaneous tissue than in visceral depots [27]. Additionally, the differentiation capability of ADSCs also depends on the characteristics of the donor, such as age, gender, and metabolic index. Older age, high BMI (>30 kg/m^2^), suffering from diabetes mellitus, or exposure to radiotherapy and endocrine therapy will decrease the proliferative and differentiation potential of ADSCs [28]. However, further research is needed to determine whether the *in vitro* and *in vivo* findings translate into clinically significant differences.

### 2.2. Isolation and Culture of ADSCs

The most widely utilized method for isolating ADSCs was first proposed by Zuk and colleagues [29]. This method involves extensive washing with phosphate-buffered saline (PBS) and digestion of lipoaspirate with 0.075% collagenase to release the stromal vascular fraction (SVF) of cells. The SVF is incubated in the medium overnight at 37°C in an atmosphere with 5% CO_2_ after a series of washes and centrifugation steps. Following incubation, the plates are extensively washed with PBS to remove residual, nonadherent red blood cells. The resulting cells are considered to be ADSCs.

Collagenase digestion remains the gold standard among the currently used methods for isolating ADSCs, although other enzymes, such as trypsin, clostripain, and dispase, can also be used [30]. A recent study suggested that, even though trypsin-digested and collagenase-digested ADSCs present similar adipogenic differentiation and proliferative ability, the osteogenic differentiation potential of the trypsin-treated cells is up to sevenfold higher [31]. Despite the widespread use of the above-mentioned methods for isolating ADSCs, enzymatic digestion-based methods have many disadvantages. The use of enzymes may alter or disrupt cell viability and surface antigens, which may reduce ADSC regenerative potential [32, 33], while question marks also remain regarding whether residual enzyme activity can affect safety. Consequently, an increasing number of studies have explored economical enzyme-free methods for ADSC isolation, including new mechanical methods [34, 35] or techniques that do not rely on enzymatic activity or centrifugation [36].

Although ADSC culture methods can vary across laboratories, a typical culturing condition comprises a monolayer of cells cultured with 10% fetal bovine serum (FBS) and 1% antibiotics at 37°C and 5% CO_2_ [37]. Although effective, the use of FBS in cell culturing processes is highly discouraged by regulatory agencies due to the potential risk of the transmission of xenogeneic infectious agents and immunization [38]. Many researchers propose alternative protocols, such as the use of human platelet lysate [39], which shows equivalent results in relation to the typical FBS-related methodologies. Additionally, platelet-rich plasma (PRP) also presents an efficient alternative supplement for ADSC proliferation [40]. Atashi et al. studied the capacity of autologous nonactivated PRP (nPRP) or thrombin-activated PRP (tPRP) on ADSC proliferation compared with 10% FBS. The final results revealed that nPRP possessed stronger proliferation-promoting effects than FBS or tPRP without changing the ADSC phenotype and chromosome status.

### 2.3. Identification of ADSCs

The presence of ADSC characteristics is commonly evaluated by flow cytometric analysis of cell surface markers [37], and the International Society for Cellular Therapy (ISCT) and the International Federation for Adipose Therapeutics and Science (IFATS) specify three minimal criteria for defining ADSCs: (1) cells must be plastic-adherent; (2) they must express CD73, CD90, and CD105 and lack the expression of CD14, CD11b, CD45, CD19, CD79, and human leukocyte antigen-DR (HLA-DR); and (3) they must have the potential to differentiate into preadipocytes, chondrocytes, and osteoblasts [37]. The ISCT also proposed that MSCs should lack the expression of CD117, CD14, CD11b, CD34, CD45, CD19, and CD79; nevertheless, the definitive markers that can effectively discriminate ADSCs remain controversial [41] (Table 1). Numerous studies have confirmed that ADSCs can express CD34 [42]. Compared with late passage ADSCs, early passage cells express higher levels of CD117, HLA-DR, and CD34 [43]. Although there are several differences among isolation and culture procedures, the immunophenotype remains consistent across laboratories. The immunophenotype of ADSCs is >90% identical to that of BM-MSCs [44]. Similar to BM-MSCs, ADSCs show uniformly positive expression of the surface antigen markers CD90, CD73, CD105, and CD44 but are negative for CD45 and CD31 [45]. Flow cytometric analysis has shown that ADSCs express CD13, CD29, CD34, CD36, CD49d, CD73, and CD133 [46]. More specifically, BM-MSCs lack the expression of CD34 and CD49d, and only ADSCs express these markers [47].

Furthermore, the detection and identification of the multiple differentiation of ADSCs are necessary. The osteogenic, chondrogenic, and adipogenic differentiation in ADSCs could be detected by the ALP assay, oil red staining, and GAG analysis [48, 49]. The real-time PCR assay may also be useful in the detection of neuron-like cells, hepatocytes, and myocytes, which are derived by differentiation of ADSCs [50]. The extraordinary characteristics of ADSCs endow them with considerable potential for use in tissue engineering and regenerative medicine. However, a standard definition of harvesting and processing techniques has yet to be established. More extensive studies are required to set a standard protocol, which would contribute significantly to the development of adipose tissue engineering.

### 2.4. Paracrine Secretion by ADSCs

Many studies have summarized the secretory profiles of ADSCs, which were assessed using enzyme-linked immunosorbent assays or related techniques. The proangiogenic and cardioprotective effects of ADSCs have been attributed to the production of growth factors, including fibroblast growth factor 2 (FGF-2), vascular endothelial growth factor (VEGF), hepatocyte growth factor (HGF), and insulin-like growth factor 1 (IGF-1) [51]. Matrix metalloproteinase- (MMP-) 3 and MMP-9 are expressed by ADSCs and are vital for the higher proangiogenic activity observed in ADSCs when compared with that of BM-MSCs [52]. Therefore, if ADSCs are exposed to a focus of inflammation or ischemic injury, they will secrete growth factors and cytokines to promote healing and tissue regeneration. ADSCs also secrete high levels of factors that have a significant role in neuroprotection and differentiation, such as brain-derived neurotrophic factor (BDNF), nerve growth factor (NGF), and glial-derived neurotrophic factor (GDNF) [53]. At the level of the immune system, there is substantial evidence that prostaglandin E2 (PGE2) partially regulates some of the immunomodulatory properties of ADSCs. In response to inflammatory stimuli, ADSCs can increase the production of angiogenic factors such as VEGF, HGF, and IGF-1 as well as that of hematopoietic/inflammatory factors such as macrophage-colony stimulating factor (M-CSF), granulocyte-colony stimulating factor (G-CSF), interleukin- (IL-) 6, and tumor necrosis factor (TNF) [54]. These findings demonstrate that both ADSCs and BM-MSCs can suppress the immune response by suppressing peripheral blood mononuclear cell proliferation and the differentiation of immature monocyte-derived dendritic cells. However, higher levels of cytokine secretion by ADSCs induce stem cells to increase the release of immunomodulatory factors [55] such as IL-10, PGE2, galectin-1, and galectin-3. Studies have shown that PGE2 and IL-10 [56] can suppress the maturation of dendritic cells and helper T cells following their activation, thereby limiting inflammation, while transforming growth factor-beta (TGF-*β*) can accelerate the premature differentiation of T helper cells into T regulatory cells [57].

## 3. Regenerative Medicine Based on ADSCs

### 3.1. Bone Regeneration

**Figure 2:**
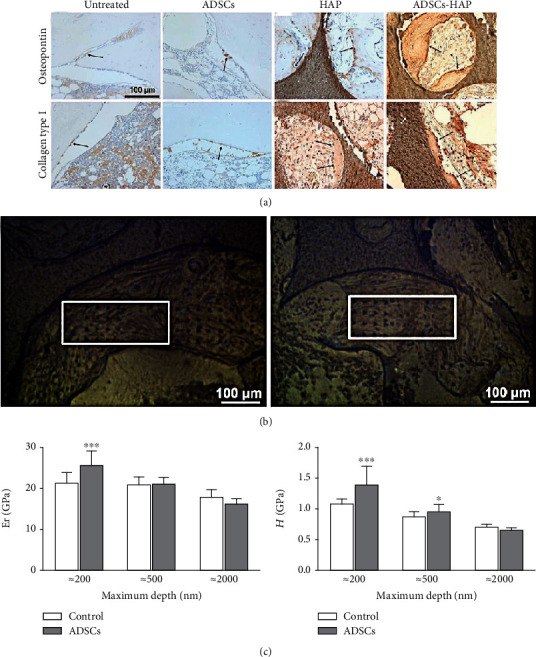
Results of the expression of bone markers and mechanical properties of scaffold construct (a). Expression of osteopontin and collagen type I in sections of decalcified tibial samples (b). Locations of the indentation experiments on the empty hydroxyapatite disk (control) and the ADSC-hydroxyapatite disk (ADSCs) (c). The reduced modulus Er and hardness *H* values of the two groups at the three loads were investigated. Adapted from a previous study [58], with permission.

Bone tissue engineering (BTE) is an optimal therapeutic approach for reconstructive surgery to repair critical-size bone defects and improve patient quality of life following high-energy trauma, malformations, osteomyelitis, and tumor resection. Osteoprogenitor seeding cells, combined with an appropriate scaffold and bioactive factors, are crucial for BTE. ADSC-based strategies for bone regeneration are widely used as ADSCs can differentiate into osteoblasts. In this study, the immunohistochemical analyses presented the new immune-positive bone tissue and bone trabeculae in the hydroxyapatite (HAP) group and HAP+ADSC group (Figure 2(a)). Meanwhile, the residual indents caused by nanoindentation testing at the maximum force of 50 mN were clearly visible (Figure 2(b)). Moreover, the ADSC-seeded scaffold construct was found to be much stiffer and harder than the unseeded scaffold (Figure 2(c)) [58].

To ensure the efficacy of ADSC-based therapeutic for bone regeneration, the related factors should be highly valued. Firstly, the subpopulations of ADSCs could affect osteogenic performance, such as pericytes and adventitial cells, which could improve angiogenic and osteogenic differentiation ability. Some authors defined CD146+ CD34- CD45- as pericytes and CD146- CD34+ CD45- as adventitial cells; these cells are isolated from multiple organs, including adipose tissue, possessing the capacity to differentiate into osteoblasts and displaying a synergistic function to promote bone healing [59]. In their trials, the sorted pericytes formed significantly more bone in comparison with unsorted cells.

Bone regeneration also involves a complex interaction between ADSCs and biological factors. The concentration of bioactive factors and the degree of tropism associated with the differentiation medium will affect the osteogenic potential of ADSCs [60]. ADSCs release growth factors that promote angiogenesis and enhance bone formation, including PDGF, VEGF, FGF-2, MMP, and bone morphogenic protein- (BMP-) 2 [61]. Recently, Yanai et al. showed that the expression level of BMP-2 can be enhanced *via* augmenting extracellular calcium concentrations; this increase activates the calcium-sensing receptor (CaSR), leading to a transient increase in intracellular calcium concentration and the stimulation of the calcium/calmodulin-dependent nuclear factor of activated T cell signaling pathway [62]. Another study reported that miRNA-375 promotes ADSC osteogenic differentiation through the Yes-associated protein 1/DEP domain containing mTOR interacting protein/protein kinase B (YAP1/DEPTOR/AKT) regulatory network [48]. Additionally, the inductive medium (ascorbic acid and dexamethasone) also affected the production of both osteogenic and angiogenic factors [63].

To be ideal bone graft substitutes, scaffolds must be biodegradable and biocompatible and exhibit strong osteoinductive properties. To date, ADSCs have been employed for BTE using several types of organic or inorganic scaffolds, including decellularized matrices, ceramics (e.g., HAP, tricalcium phosphate, coralline-derived HAP, calcium sulfates, glass ceramics, calcium phosphate-based cement, and bioglass), synthetic polymers and hybrid scaffolds (e.g., polylactic acid (PLA), polyglycolic acid (PGA), copolymer poly(lactic acid-co-glycolic acid) (PLGA), and polycaprolactone (PCL)), and natural polymers (e.g., fibrin, collagen, gelatin, and silk) [64]. Different scaffolds have different advantages and disadvantages. The composition of synthetic polymers can be controlled, thereby reducing the risk of toxicity, immunogenicity, and the favoring of infection. However, the hydrophobicity of synthetic scaffolds can lead to problems in cell adhesion and infiltration [65, 66]. Ceramics exhibit osteoconductive properties and can bind directly to the bone under certain conditions [67]. However, owing to their slow degradation rate and low mechanical strength, they are not suitable for use as a loading scaffold alone [68, 69]. Hybrid scaffolds are composed of organic and inorganic materials that can gradually degrade without generating toxic byproducts. The type of structure acquires multiple functionalities with appropriate mechanical and thermal properties as well as structural stability [70]. Mazzoni et al. reported that porous hydroxylapatite/collagen composite biomaterials have excellent osteoinductive properties and show good biocompatibility [71].

The type of mechanical support also will affect osteogenic potential. Optimal porosity plays an essential role in directing the cells to grow into the desired physical form and to support the vascularization of the ingrown tissue [72]. Notably, total porosity and bone surface area are the main factors that must be controlled. Pore diameters of 150 mm were shown to improve endothelial cell function, as evidenced by the promotion of cell adhesion and migration, increased cell proliferation, and the enhanced expression of platelet-endothelial cell adhesion molecules (PECAMs) and VEGF [73]. A typical porosity of 90% and a pore size of at least 100 mm are known to be necessary for cell penetration and the proper vascularization of bone tissue. The mechanical properties of the scaffold are also affected by stiffness. Nii et al. used a poly(ethylene glycol) diacrylate platform to culture ADSCs and examine mineralization and osteocalcin gene expression and found that intermediate stiffness and a low concentration of fibronectin could increase osteocalcin gene expression by over 130-fold [74].

Based on *in vitro* experiments and preclinical studies, the capability of ADSCs to promote bone regeneration has been verified in clinical studies. A case report describing the repair of a posttraumatic calvarial defect using autologous ADSCs in a seven-year-old child was the first clinical study to be published on ADSC-repaired bone defects. Owing to the limited amount of autologous cancellous bone available from the iliac crest, the ADSCs were engrafted onto the calvarial defect. The postoperative course was uneventful, and computed tomography scans showed new bone formation and near-complete calvarial continuity 3 months after the reconstruction [75]. Current clinical ADSC therapies for bone regeneration have demonstrated promising results for craniofacial [76, 77] and lone bone defects [78]. Although *in vitro* experiments, preclinical trials, and clinical studies have confirmed the osteogenic differentiation ability of ADSCs, further investigations are still needed to standardize the procedures for the use of ADSCs in bone regeneration.

### 3.2. Cartilage Regeneration

**Figure 3:**
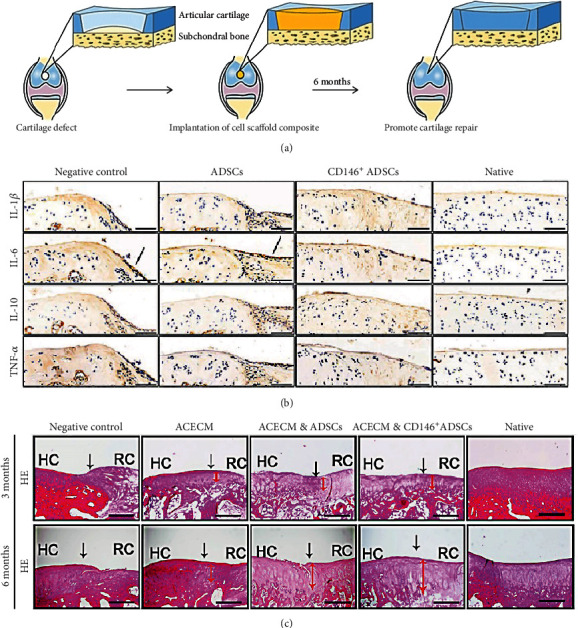
Flow chart of the experimental steps for long-term cartilage repair in rabbits (a). Immunohistochemical staining of interleukin- (IL-) 1*β*, IL-6, IL-10, and tumor necrosis factor (TNF). Black solid arrows denote the positive expression of IL-6 in the repair interface (b). Histological analysis of the cartilage defect after 3 and 6 months by hematoxylin and eosin (H&E) staining. Black solid arrows denote the repair interface. Red solid arrows denote the depth of the repaired cartilage (c). HC: host cartilage; RC: repaired cartilage. Adapted from a previous study [84], with permission.

Cartilage injury is a major cause of disability worldwide owing to the weak self-healing ability of cartilage tissue [79]. Currently, the clinically applied cartilage repair approaches include microfracture [80], subchondral drilling [81], and autologous chondrocyte implantation [82]; however, the limited availability and substantial associated donor site morbidity restrict their application [83]. The emergence of ADSC-based cartilage tissue engineering has received particular attention. CD146 is not a specific osteogenic marker in ADSC subpopulations; indeed, there is evidence that a CD146+ subset of ADSCs also has chondrogenic differentiation potential, as well as inflammation-modulating properties (Figures 3(a)–3(c)) [84]. Interestingly, CD146-negative subsets also have a similar cartilage differentiation ability [85]. The cell-biomaterial correlative structure established between surface receptor and adhesion molecules on the surface of materials enhanced the chondrogenic differentiation of ADSCs into articular chondrocytes [86]. On the other hand, the expression of the CD73, CD90, CD105, and CD106 markers is also necessary for ADSC differentiation into cartilage [87].

In addition to the use of specific subpopulations of ADSCs, biological factors are also indispensable for enhancing cartilage formation. *In vitro* studies have demonstrated that ADSCs can differentiate into chondrocytes when they are cultured with IGF-1, TGF-*β*, or BMP, and these chondrocytes express the same type II collagen as mature chondrocytes [49, 88, 89]. Several TGF-*β* members such as TGF-*β*1, 2, and 3 are known to possess good chondrogenic differentiation potential [90]. An induction medium containing a combination of TGF-*β*3 and BMP-6 has shown better chondrogenic potential than that containing TGF-*β*3 alone [91]. Moreover, L-ascorbic acid and PRP can maintain the survival of ADSCs and improve their expected chondrogenic function when delivered at an appropriate concentration [92]. Current studies have focused on the efficacy of PRP in cell differentiation and proliferation as it contains high concentrations of PDGF, TGF-*β*, IGF, VEGF, and EGF [93]. TGF-*β* positively regulates the transcription of chondrogenesis-related genes, including SRY-box transcription factor 9 (*SOX9*), through SMAD phosphorylation [94]. The SOX9 protein, one of the earliest chondrogenic markers, is essential for the expression of collagen type II [95]. Liao et al. discovered that the overexpression of SOX9 enhanced BMP2-induced chondrogenic differentiation and inhibited the osteogenic differentiation of MSCs [96].

Osteoarthritis (OA) is a progressive degenerative joint disease characterized by the deterioration of articular cartilage and pathological changes in the adjacent subchondral bone [97]. Current conventional treatments (physical therapy, glucosamine, chondroitin sulfate supplementation, and arthroscopic surgery) or surgical therapies (abrasion arthroplasty, subchondral drilling, and microfracture) are aimed at alleviating pain and enhancing joint function; however, they are limited by their low efficacy [98], and intra-articular (IA) injection of ADSCs to repair damaged cartilage has potential as a suitable alternative. Recently, Spasovski et al. suggested that the IA injection of a proposed dose of ADSCs may be a safe and efficient method for use in the treatment of osteoarthritis. During a 6-month follow-up, they found that the clinical symptoms had improved following an IA injection of ADSCs [99].

Despite the marked clinical efficacy of IA, the dose and timing of ADSC injection are important. In a study aimed at evaluating the safety and therapeutic potential of autologous human adipose-derived mesenchymal stem cells in patients with osteoarthritis, 18 patients with knee osteoarthritis were enrolled and divided into three dose groups: low dose (1.0 × 10^7^ cells), middose (2.0 × 10^7^), and high dose (5.0 × 10^7^); clinical, radiological, and histological parameters were evaluated with 96 weeks of follow-up. The high-dose group exhibited better pain relief and greater improvement in knee function than the other two groups [100]. Several studies have indicated that the inhibitory effect of ADSCs is affected by the stage of OA. In a mouse model, a single injection of ADSCs into the knee during the early stage of OA can inhibit synovial thickening, the formation of enthesophytes associated with ligaments, and cartilage destruction. However, no effect was observed in the late stage of the disease [101]. In addition, swelling of the injected joints is frequently observed and is thought to be associated with the survival rate of the ADSCs [102]. Directly injected cells usually have limited cell retention and survival rates, especially in large cartilage lesions. Koh and colleagues reported that ADSCs seeded in scaffolds may have better viability, preservation, and aggregation [103]. To improve the efficacy of this procedure, as well as the comfort of the patients, appropriate cell-loaded scaffolds should be developed for treating patients with large cartilage defects.

The 3D structure of loaded ADSCs is a key for promoting the recovery of joint cartilage, and the materials, pore size, and rigidity of the scaffold must all be taken into consideration. Natural materials should favor cell adhesion and exhibit enhanced mechanical support and biodegradability [104]. Type I collagen is an appropriate scaffold as it induces low inflammatory responses and also has excellent cell compatibility. Recent findings have underlined that 3D collagen scaffold culture combined with PDGF and insulin promotes the chondrogenic differentiation of ADSCs [105]. Studies have confirmed that hydrogel-based scaffolding systems also allow for the creation of high-quality engineered cartilage but may exhibit inferior mechanical properties [106]. The replacement of a natural scaffold with a synthetic material allows the artificial adjustment of the pore size and stiffness of the structure. Based on the above characteristics, Yin et al. concluded that a TGF-*β*1-immobilized PLGA-gelatin scaffold seeded with ADSCs considerably enhanced the quality of the tissue-engineered cartilage [107]. The effect of scaffold pore size on chondrogenesis should also be taken into account. The proliferation and chondrogenic differentiation of stem cells are affected by scaffold porosity [108]. Scaffolds with smaller pore sizes (90–250 *μ*m) are better for preserving cell adhesion and proliferation and also allow for higher expression levels of collagen, aggrecan, and type II collagen [109].

Sometimes, the cause of a knee injury may be a defect in the meniscus, and a degenerating meniscus leads to instability and a low level of nutrient supply to the cartilage. Intra-articular injection of stem cells can promote meniscus regeneration, and the immature meniscus will protect cartilage [110]. In conclusion, it is important to establish a therapeutic specification and provide suitable, patient-specific solutions.

### 3.3. Nervous System Regeneration

Studies have shown that ADSCs can differentiate into neurons, endothelial cells, and Schwann cells [111] and exhibit higher levels of neural marker expression and a faster proliferation rate than other stem cells [112]. The neural differentiation of ADSCs involves a complex regulatory network. ADSCs are known to release a range of neurotrophic factors, including NGF, BDNF, GDNF, FGF, and IGF-1, which are vital for the healing and regeneration of damaged nerves [113, 114]. Vascularization also plays a pivotal role in nerve healing by sustaining cell survival and promoting cell proliferation [115]. Furthermore, ADSCs also regulate antiapoptotic functions [116].

Controlling the inflammatory response could be thought of as another element in neural repair. TNF*α*-stimulated gene-6 (TSG-6) is a component of the negative feedback loop secreted by ADSCs [117]. It can reduce signaling in the resident macrophages and thereby modulates the cascade of proinflammatory cytokines. A growing body of evidence has confirmed the therapeutic potential of ADSCs in rebuilding the central nervous system and peripheral nervous system.

### 3.4. Central Nervous System (CNS) Regeneration

**Figure 4:**
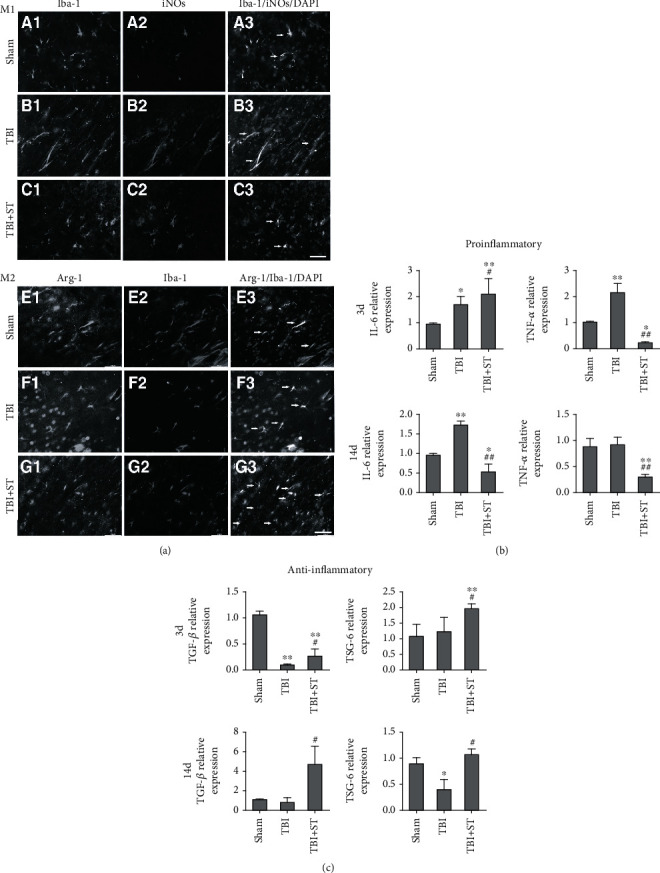
Double immunostaining with anti-iNOS and anti-Iba-1 antibodies to identify M1 and M2 microglia in the cortex within 1 mm of the lesion in the sham, TBI, and TBI+secretome of ADSCs (TBI+ST) groups 7 days after traumatic brain injury (TBI) (a). Cytokine expression levels at 3 and 14 days after TBI were evaluated by qPCR (b, c). iNOS: inducible nitric oxide synthase; Iba-1: ionized calcium-binding adaptor molecule 1; Arg-1: arginase 1. Adapted from a previous study [119], with permission.

Several animal models of SCI and TBI have been developed to evaluate the efficacy and safety of ADSC-based therapy. Primary acute injury results mainly from the immediate external force exerted on the brain, whereas secondary injury occurs over time through a cascade of biochemical activation that leads to neuroinflammation and neurodegeneration; the latter is also the primary mechanism associated with subacute and chronic phases [118]. Current therapies for TBI focus primarily on suppressing the secondary insult. Xu et al. found that ADSCs can modulate TBI-induced neuroinflammation and subsequent secondary injury by increasing the ratio of M2 (anti-inflammatory) to M1 (proinflammatory) microglia (Figure 4(a)). The M1-related proinflammatory cytokines IL-6 and TNF and the M2-related anti-inflammatory cytokines TGF-*β* and TSG-6 have changed accordingly (Figures 4(b) and 4(c)) [119]. Additionally, ADSC-derived exosomes can inhibit the activation of microglia by downregulating nuclear factor kappa-B and the mitogen-activated protein kinase (MAPK) pathway and can also reduce the cytotoxicity associated with activated microglia [120]. Neuronal degeneration and blood vessel damage following a traumatic wound can induce inflammation, followed by the loss of neurons and oligodendrocytes. Therefore, controlling the inflammatory response after injury may have potential as a therapeutic option [121]. Yin and colleagues seeded ADSCs on acellular spinal cord scaffolds and demonstrated that this model enhanced functional recovery in spinal cord-injured rats by promoting axon regeneration and reducing reactive gliosis [122]. An ongoing multidisciplinary clinical trial also presents positive results [123]. In this trial, ADSCs are intrathecally injected at the L3–4 level. The subjective (physical therapy and occupational therapy reports) and objective (International Standards for Neurological Classification of Spinal Cord Injury scores) measures showed different degrees of improvement. For neurological disorders, such as amyotrophic lateral sclerosis [124], Alzheimer's disease (AD) [125], Huntington's disease [126], and Parkinson's disease (PD) [127], the treatment efficacy of ADSCs was confirmed in some animal and cell models. Many clinical trials are underway to test the efficacy and safety of ADSC-based treatment in AD and PD patients (ClinicalTrials.gov Identifier: NCT03117738 and NCT02184546).

### 3.5. Peripheral Nervous System (PNS) Regeneration

**Figure 5:**
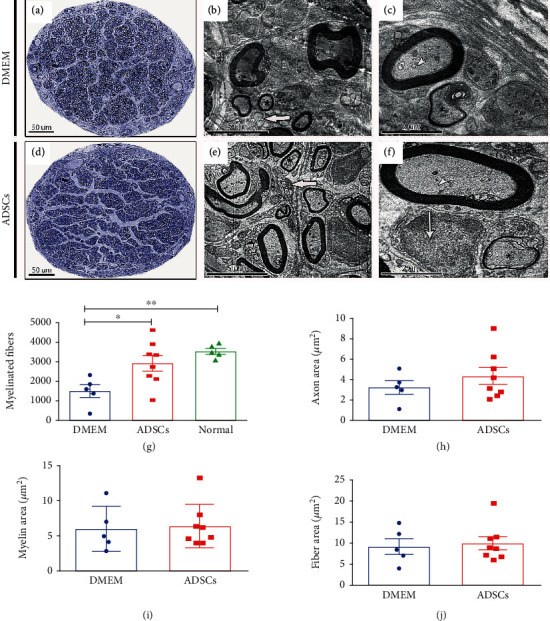
Representative images of semithin cross-sections of the regenerating sciatic nerve in the Dulbecco's modified Eagle's medium (DMEM) and ADSCs (a–c). Electron micrographs of a regenerating sciatic nerve in the transverse plane (b, c, e, f). Graph showing the total number of myelinated fibers in the sciatic nerve for all the groups (g). Quantitative morphological analyses of the axon area, fiber area, and myelin area in the regenerating sciatic nerve (h–j). Adapted from a previous study [132], with permission.

Peripheral nerve injury (PNI) is a complicated, multifactorial disorder with varying degrees of severity. During peripheral nerve repair, Schwann cells are the main factors promoting axonal regeneration in distal nerve stumps [128]. Recent studies have reported that ADSCs can differentiate into Schwann cells and facilitate native Schwann cell activity [129]. To bridge nerve defects, scientists have focused on nerve conduits and acellular nerve grafts combined with ADSCs. For conduit scaffolding, the tube was initially composed of silicon; however, highly biocompatible materials, such as autogenous vein nerve conduits, allografts, PGA, PCL, and collagen, are now used in tubes [130]. PGA-collagen conduits have been tested in a 15 mm gap model to compare the regenerative nerve effects of conduits combined with or without ADSCs and resected nerve [131]. PLA conduits and cell therapy with ADSCs lead to a better functional and morphological recovery after sciatic nerve transection. Nerves in the ADSC experimental group showed a greater number of myelinated fibers and better tissue organization with well-defined fascicles compared with the Dulbecco's modified Eagle's medium (DMEM) experimental group (Figures 5(a)–5(f)). The total number of myelinated fibers was significantly greater in the ADSCs and normal group compared with that in the DMEM group (Figure 5(g)). Meanwhile, a quantitative morphological analysis of the axon area, fiber area, myelin area, and G-ratio in the regenerating sciatic nerve did not show statistically significant differences among the experimental groups (Figures 5(h)–5(j)) [132]. However, to date, experiments have been conducted using small animals, and future evaluations will inevitably have to include larger animals to allow the progression toward clinical applications.

### 3.6. Myocardium Regeneration

Cardiovascular disease (CVD) is the leading cause of death globally and can lead to ischemia in critical regions, as well as myocardial necrosis. Ischemic heart disease, particularly myocardial infarction (MI), is a typical type of CVD that can cause heart failure [133]. ADSC therapy has been widely investigated as a prospective treatment for MI in preclinical and clinical trials. The mechanics of the therapeutic application of ADSCs in CVD can be classified into three categories: the differentiation of ADSCs into cardiomyocytes [134]; supplying a large amount of antiapoptotic, angiogenic, and anti-inflammatory factors [135, 136]; and preventing adverse cardiac remodeling by inhibiting myocardial fibrosis [137]. To date, four different transplant methods—intramyocardial injection, intravenous injection, intracoronary injection, and cell spray transplantation—have been intensively investigated. Although the effect of the intravenous injection is affected by a pulmonary first-pass effect, this method showed a beneficial influence on reducing infarct size and enhancing cardiac function and blood vessel formation [138]. For left ventricular (LV) systolic function, both intramuscular injection and intracoronary injection show a promising ability to improve the left ventricular ejection fraction (LVEF) [139]. Bobi et al. reported that intracoronary injection suppressed the apoptosis of infarcted myocardium but did not significantly change the LVEF [140]. Stem cell spray transplantation markedly attenuated left ventricular remodeling and enhanced vascular density in the infarct border area [141].

**Figure 6:**
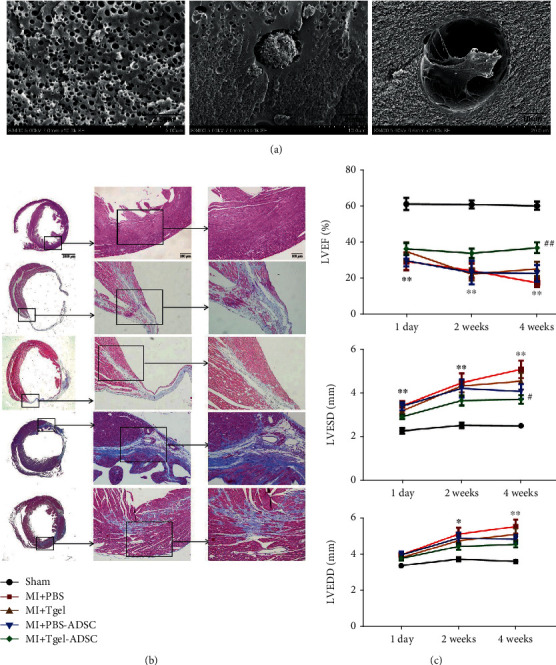
Scanning electron micrographs of Col-T gel-encapsulated ADSCs 3 days after encapsulation (a). Representative images of Masson trichrome staining of the transverse planes of heart sections (b). LVEF, LVESD, and LVEDD at 1 day, 2 weeks, and 4 weeks after myocardial infarction (c). LVEF: left ventricular ejection fraction; LVESD: left ventricular end-systolic diameter; LVEDD: left ventricular end-diastolic diameter. Adapted from a previous study [144], with permission.

The efficacy of ADSC injection into the infarcted myocardium remains limited by low survival and retention rates. Numerous attempts have been made using preconditioning and engineering strategies to overcome these hurdles. Guo et al. found that resistin-treated ADSCs intravenously injected into mice with myocardial ischemia significantly improved the LVEF, mitigated fibrosis, and reduced cardiomyocyte apoptosis [142]. The same effect was found with melatonin pretreatment [143]. The engineered ADSCs enhanced retention, increased angiogenesis, reduced the degree of fibrosis, and decreased infarct size. When compared with ADSCs alone, transglutaminase cross-linked gelatin (Col-T gel) combined with ADSCs markedly reduced the size of the myocardial fibrotic area (Figures 6(a) and 6(b)). T gel-ADSCs significantly increased the LVEF at 4 weeks after MI. Additionally, T gel-ADSCs significantly decreased the left ventricular end-systolic diameter (LVESD), but not the left ventricular end-diastolic diameter (LVEDD), at 4 weeks after MI when compared with PBS treatment (Figure 6(c)) [144]. Furthermore, the decellularized extracellular matrix created a favorable microenvironment for ADSCs in the infarct area, reducing fibrosis and increasing the LVEF [145]. Genetic modification, which can be used to enhance the secretion of stromal cell-derived factor 1 (SDF-1), IGF-1, VEGF, HGF, and FGF-2, has been extensively investigated in heart regeneration. This approach is correlated with reduced cardiomyocyte apoptosis and enhanced angiogenesis [146, 147].

Convincing evidence obtained in preclinical ADSC transplantation studies on MI has prompted several clinical trials. The APOLLO trial was a randomized, double-blind, placebo-controlled, phase I/II study (NCT00442806) to test the feasibility of using ADSC transplantation for the treatment of STEMI [148]. The results showed that ADSC infusion could improve cardiac function and perfusion defects, accompanied by a 50% reduction in myocardial scar formation. ATHENA trials I (NCT01556022) and II (NCT02052427) focused on assessing intramyocardial ADSC transplantation. In this trial, ADSC treatment promoted a marked increment in Minnesota Living with Heart Failure Questionnaire (MLHFQ) and SF-36 scores, while heart failure and angina symptoms also improved. However, no significant changes were found in the LVEF or LV volumes by echocardiography. Further detailed and comprehensive clinical trials are needed to achieve more precise and accurate benefits in delaying ventricular remodeling and heart failure development.

### 3.7. Liver Regeneration

Acute liver failure (ALF) and chronic liver disease are mainly caused by exposure to factors such as viral infection, toxins, and genetic disorders. ADSC-based therapy is a promising alternative for the treatment of these disorders. ADSCs can differentiate into several types of liver cells and secrete antiapoptotic or anti-inflammatory factors, thereby promoting the healing of liver injury [149, 150].

Ischemia-reperfusion injury (IRI) is a universal complication of liver surgery, often leading to postoperative complications and liver dysfunction. Ge et al. injected ADSCs into the liver parenchyma following partial laparoscopic hepatectomy [151]. ADSC treatment increased the activity of superoxide dismutase and suppressed the generation of both myeloperoxidase and malondialdehyde, thereby reducing oxidative stress. Additionally, ADSC treatment led to a marked decline in the levels of adverse hematological indicators, such as aspartate aminotransferase (AST), alanine aminotransferase (ALT), total bilirubin (T-BIL), and lactate dehydrogenase (LDH). A different study reported that ADSCs suppressed the level of inflammatory cytokines such as IL-1*β*, IL-6, and TNF, while enhancing the secretion of the anti-inflammatory factor IL-10 and the regenerative factors HGF and cyclin D1, thereby ameliorating the IRI-induced damage [152]. Similar hepatoprotective effects were also found in other trials [153]. In a carbon tetrachloride- (CCl_4_-) induced acute liver injury model, Yan et al. intravenously injected ADSCs to assess their effects on acute liver injury [154]. ADSC treatment reduced the serum concentrations of ALT, AST, and T-BIL and restored the liver structure and glycogen synthesis ability in the canine model animals.

**Figure 7:**
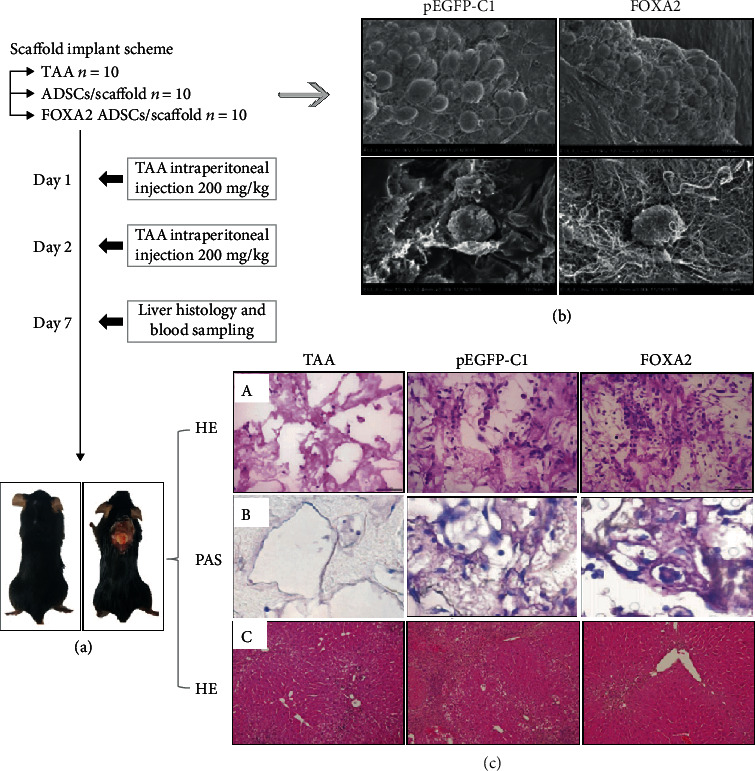
A schematic representation of the experimental design (a). Scanning electron micrographs of ADSCs in a pEGFP-C1-transfected ADSCs/scaffolds and FOXA2-transfected ADSCs/scaffolds (b). Hematoxylin and eosin (H&E) staining of the necrotic area and retrieved scaffolds (c). TAA: thioacetamide. Adapted from a previous study [158], with permission.

Liver fibrosis is a frequent outcome of chronic liver disease and is characterized by hepatocyte death, hepatic inflammation, and activation of hepatic stellate cells (HSCs) [155]. Studies to date have shown that ADSCs suppress the expression of inflammatory cytokines and the proliferation of alpha-smooth muscle actin-positive activated HSCs [156]. Hao and colleagues showed that ADSC transplantation markedly attenuated liver fibrosis by inhibiting HSC proliferation and promoting HSC apoptosis in animals with CCl_4_-induced liver fibrosis [157]. For the treatment of liver diseases, the ADSCs that are functionally reinforced through pretreatment have greater therapeutic efficacy. Forkhead box transcription factor 2- (FOXA2-) overexpressing ADSCs loaded in a PLGA scaffold markedly reduced the size of the necrotic area and improved liver function in an acute liver injury model (Figures 7(a)–7(c)). The FOXA2-overexpressing experimental group showed greater glycogen storage ability (Figure 7(c), i and ii). The necrotic area was significantly lower in the FOXA2-overexpressing ADSC/scaffold group than in the other groups (Figure 7(c), iii) [158]. ADSCs cultured in hypoxia-conditioned media induced higher expression of antioxidant enzymes and nuclear factor erythroid 2-like 2 (Nrf2), thereby protecting against reactive oxygen species-related toxicity in the injured liver [159]. Many clinical trials have been designed to confirm the efficacy and safety of ADSCs in patients with liver cirrhosis or ALF. In these trials, ADSC transplantation did not raise any safety concerns. Besides, tests that measure liver function, such as the ^13^C methacetin breath test, METAVIR score, Child-Pugh score, and MELD score, have yielded positive results [160, 161]. Combined, these findings suggest that ADSC transplantation is a promising therapeutic option for the treatment of liver injury. However, additional clinical trials with large sample size are needed to convincingly show the benefits of using ADSCs.

### 3.8. Skin Wound Healing

**Figure 8:**
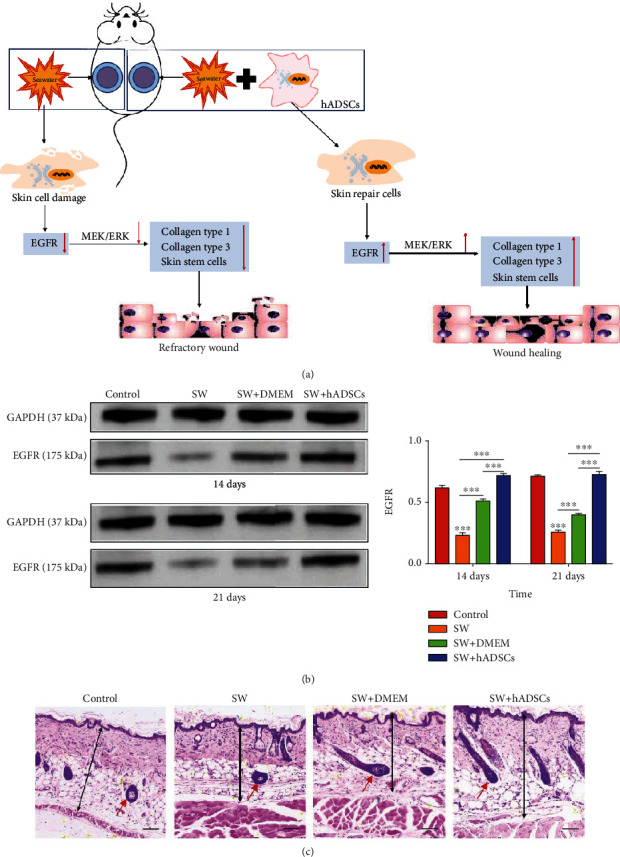
Schematic showing how seawater (SW) and adipose-derived stem cells (ADSCs) regulate wound healing through the EGFR/MEK/ERK signaling pathway (a). The EGF protein expression levels were significantly higher in the control and SW+ADSC groups than in the SW and SW+DMEM groups (b). Hematoxylin and eosin (H&E) staining for wound repair, skin thickness, and a number of subcutaneous appendages (c). The red arrow denotes a hair follicle. Adapted from a previous study [165], with permission.

Preclinical and clinical trials have recently greatly improved the use of ADSC therapy for the treatment of severe burn injuries and intractable ulcers [162], which involves the interaction of many soluble factors and the activation of multiple biological pathways. Angiogenesis-related cytokines released from ADSCs, such as G-CSF, PDGF, SDF-1, VEGF, b-FGF, HGF, MMP, IL-6, and IL-8, promote the recovery of wound blood supply [163]. ADSCs can not only enhance the migration and proliferation of fibroblasts but also inhibit collagen deposition and the expression of *α*-smooth muscle actin in hypertrophic scar fibroblasts [164]. ADSCs differentiate into skin stem cells and promote the accumulation of autologous skin stem cells via the epithelial growth factor receptor/methyl ethyl ketone/extracellular regulated protein kinase (EGFR/MEK/ERK) pathway to accelerate wound healing (Figures 8(a)–8(c)). Xiong et al. established a wound model of seawater (SW) immersion and compared it with normal wound healing. The results showed that the protein expression level of EGF was significantly higher in the control and the SW+ADSC groups than in the SW group or the SW+DMEM group (Figure 8(b)). Microscopic observations of wound sections showed that the skin in the SW and the SW+DMEM groups was significantly thinner than that in the control and SW+ADSC groups (Figure 8(c)) [165]. During wound healing, a reduction in wound inflammation is associated with a switch in macrophage polarization from a proinflammatory (M1) to a prorepair (M2) phenotype [166]. However, the examination of local ADSC injection always revealed reduced cell viability, which ensued from shear stress during the treatment.

ADSCs combined with a scaffold substantially improve the proliferative, differentiation, and paracrine signaling abilities of ADSCs. Li et al. discovered that ADSCs seeded on a collagen 3D scaffold could better differentiate into keratinocytes and epithelial cells than those seeded on a two-dimensional niche [167]. An *in situ* formed hydrogel system that could easily cover irregularly contoured burn wounds significantly enhanced neovascularization, accelerated wound closure, and reduced scar formation [168]. An acellular dermal matrix (ADM) combined with ADSCs attenuated inflammation in diabetic wounds and promoted wound healing. Meanwhile, immunohistochemical staining following ADM-ADSC treatment showed increased expression of EGF, Ki-67, and prolyl 4-hydroxylase and reduced expression of CD45 [169]. Ding et al. utilized Bcl-2-modified ADSCs embedded within collagen scaffolds in the treatment of diabetic wounds. This frame significantly improved wound healing, promoted neovascularization, and shortened healing time compared with the control group [170]. ADSC-based cell-free therapy and scaffold-free culture systems for repairing wounds have attracted a great deal of attention. ADSC-derived supernatants stimulate wound healing by increasing the proliferation of fibroblasts, endothelial cells, keratinocytes, and cells of human skin origin [171]. A scaffold-free culture system, called adipose-derived stem cell sheets, could inhibit CCL2 release and macrophage recruitment *via* secreting greater amounts of C1q and TNF-related 3 (C1QTNF3) in the wound area. Moreover, no transplanted ADSCs were found in the fourth week, thereby reducing the undesirable long-term side effects associated with ADSC transplantation [172]. ADSCs have also proved beneficial for chronic radiation skin injuries and ischemia-reperfusion injuries of the flap [173–175].

Compared with animal experiments, comparatively few clinical trials have been performed to evaluate ADSC treatments. Jo et al. used ADSC transplantation to repair facial skin defects in four patients and reported that the defects were rapidly covered over by the patients' regenerated tissue [176]. ADSC therapy was also effective and safe when used for the treatment of 10 cases of decade-long radiation injuries [175]. In contrast, a clinical study [177] reported that a single treatment with autologous fat grafts was insufficient to ameliorate mature pediatric burn scars, although this may have been due to the small sample size. This indicates that more accurate and rigorous trials are needed to assess the therapeutic effects of ADSCs on wound healing.

### 3.9. Other ADSC-Based Treatment Modalities

In the past few years, a wide variety of methods, particularly drug therapies, have been proposed as treatments for eye disorders. Nevertheless, there is still a lack of effective treatments for corneal injury and retina or optic nerve degeneration, and ADSC transplantation has increasingly been used for this purpose. ADSC transplantation accelerated recovery from corneal epithelial damage, as evidenced by the proliferation of corneal epithelial cells, reduced levels of inflammation-related cytokine levels, and increased numbers of M2 macrophages [178]. To date, the feasibility of using ADSCs for stabilizing the retinal microvasculature has been conclusively established in the diabetic retinopathy model [179].

Additionally, the ADSC-loaded collagen sponge is an effective strategy to repair the tracheal defect and recover the motility function of cilia [180]. Similarly, studies have found that ADSCs seeded onto an RNA-bladder acellular matrix graft scaffold could promote bladder regeneration [181]. Current evidence supports the possibility that ADSC-based therapeutic is an important site of tissue regeneration.

The details of the aforementioned studies on animals and clinical studies on humans are summarized in Tables 2 and 3.

## 4. The Potential Risk for Tumor Invasiveness Associated with ADSC-Cancer Cell Interactions

**Figure 9:**
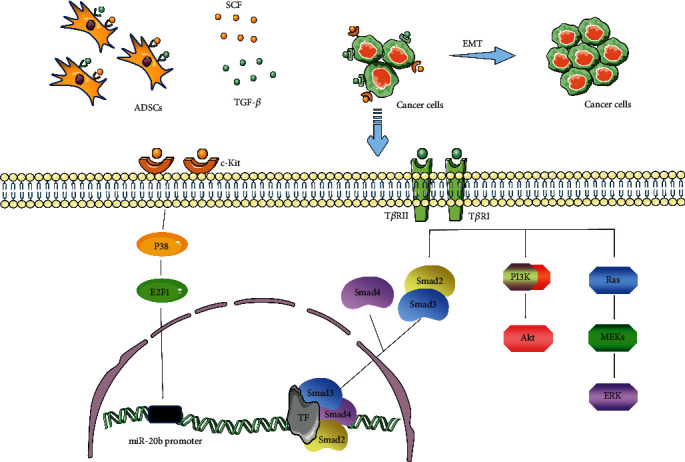
ADSCs promote the migration, invasion, and mesenchymal-epithelial transformation of cancer cells by secreting TGF-*β* and SCF. TF: transcription factor; T*β*R: TGF-*β* receptor.

Despite the large number of preclinical and clinical studies reporting the potential of stem cells to act as an “off-the-shelf” therapy for the repair and regeneration of damaged tissues, the clinical application of ADSCs remains limited. Several studies have demonstrated that the proliferative and invasive ability of breast cancer cells is increased following interaction with ADSCs. Cancer stem cells, also called tumor-initiating cells, represent a subpopulation of cancer cells displaying long-living, drug-expelling, and antiapoptotic properties [182]. Chan et al. found that hybrids produced through the spontaneous fusion of ADSCs and breast cancer cells express markers characteristic of breast cancer stem cells [183]. Additionally, the expression of HIF-1*α*/VEGF and the metastasis of breast cancer cells were induced *via* the downregulation of miR20b by ADSC-released stem cell factor (SCF), and this process was dependent on the activation of the c-Kit/p38-MAPK/E2F1 signaling pathway [184] (Figure 9). ADSCs are associated with the activation of epithelial to mesenchymal transition (EMT), another crucial step in the switch toward a more invasive phenotype. ADSCs can stimulate the expression of EMT-associated transcription factors, likely through TGF-*β*/SMAD-dependent and SMAD-independent phosphatidylinositol 3 kinase (PI3K)/AKT signaling pathways [185] (Figure 9).

The findings of the procarcinogenic role of ADSCs in laboratory studies appear to be contradictory to those of clinical reports. In the RESTORE-2 trial, the 67 enrolled patients were treated with ADSCs for the reconstruction of postoperative breast defects [186]. No treatment-related serious adverse events or local cancer recurrences were reported during the follow-up. Clinical data on the oncological safety of ADSCs are predominantly derived from female breast cancer patients, while follow-up times have been sufficiently long. Thus, further clinical studies are needed to determine whether ADSC-based regenerative therapy can be safely used for the treatment of other disorders.

## 5. Current Challenges and Future Directions

The application and development of ADSC therapy present more systematic and professional theoretical support for tissue engineering and regenerative medicine: (1) abundance and easy access, (2) immunomodulatory and anti-inflammatory effects, (3) autocrine and paracrine functions through the generation of chemokines and growth factors, and (4) the ability to differentiate into damaged tissue- and organ-specific cell types. However, ADSCs are not available as a ready-to-use product, and some key challenges remain.

Immunoreactivity is one of the greatest challenges. During ADSC culture, 10–20% FBS or calf serum is commonly used; however, the risk inherent to animal-derived products remains a concern. Contamination with viruses, prions, mycoplasmas, or unidentified xenogeneic proteins from animal-derived serum has the potential to cause immunological reactions in patients. In addition, xenobiotic growth factors may disturb ADSC differentiation and proliferation. Therefore, to avoid these risks, serum-free or xeno-free culture media without animal serum should be developed.

Genetic modification is a widely used tool for enhancing repair efficiency. Virus-associated gene transfection has been the mainstay for gene therapy to extend the functions of ADSCs. However, this procedure is inevitably associated with safety concerns, including immune reactions and vector-mediated genotoxicity. The latter may manifest as inflammation, insertional mutagenesis, and activation of protooncogenes [187]. Oncogenesis primarily occurs due to promoter insertion, promoter activation, or truncation of gene transcripts. Despite years of research and numerous clinical trials, only two gene therapy treatments, Glybera and Strimvelis, have been approved for clinical use, indicating that the choice of preclinical and clinical trial populations is important to ensure efficacy and safety.

Hladik et al. indicated that the ability of MSCs to recognize DNA double-strand breaks is gradually lost after long-term culture [188]. Additionally, the frequency of cytogenetic alterations increases in aged cells, resulting in chromosomal instability. Notably, impaired DNA damage responses and chromosomal instability may increase the risk of tumorigenesis [189]. However, ADSCs are typically considered to be stable in long-term culture *in vitro* compared with cells derived from other sources. Li et al. found that chromosomal aberrations can be detected after 20 culture passages, while the gene expression levels of p53 and telomerase reverse transcriptase remain stable at all passages [190]. This is still a controversial issue with ADSC transformation, and further experiments are needed to clarify this concern.

Taking account of the expense and complexity of the regulatory problems associated with ADSCs, it is evident that a large part of physicians are hesitant to perform any stem cell supplemented transfer operation procedures [27]. Meanwhile, automated devices for isolating ADSCs are classified into class III medical devices by the Food and Drug Administration (FDA), which cannot be approved for clinic application. Besides, the FDA stipulates that ADSC transplantation must be minimally manipulated, enzyme-free, and used in the same surgical procedure. Thus, an enzyme-free, cost-effective, and reproducible manufacturing of high-quality ADSCs for clinical use is desperately needed.

In addition to the aforementioned challenges, the biomaterials and their impact on ADSC in tissue engineering also needed more long-term *in vivo* experiments. Although biomaterials are biocompatible, most parts of them are extracted from animals and may prompt an immune response in the long term [10]. Moreover, with the prolongation of the culture time in the body, the biomaterials will be degraded and the fraction may serve as host antibodies eliciting the robust immune response. In this sense, further prospective studies investigating the safety of the biomaterials should be carried out, before application in human patients.

In summary, further preclinical and clinical studies are needed to determine whether ADSC-based therapies can fulfill expectations and be used to reconstruct damaged organs or tissues to treat diseases for which current treatments are ineffective.

## 6. Conclusion

The emergence of ADSC therapy provides a novel means for tissue regeneration. Numerous clinical and preclinical studies have demonstrated the vital role of ADSCs in reconstructing and repairing target organs, such as bone, cartilage, myocardium, liver, nervous system, and skin. However, many safety issues need to be urgently addressed, from the preparation of ADSCs to their application. Furthermore, additional researches are required to identify appropriate scaffolds and potent inducing bioactive factors to provide an optimal microenvironment for ADSC proliferation and differentiation, and long-term studies are needed to ensure the implant-tissue interactions, resorption, and hierarchical structure and finally to turn them into a clinically viable method. Due to the significant differences between preclinical studies and clinical trials, the oncogenicity of ADSC differentiation warrants further research. Despite current challenges, the great pace of progress in this field suggests that ADSC-based approaches will play increasingly important roles in regenerative medicine.

## Figures and Tables

**Table 1:** Potential surface markers for the identification of ADSCs.

Surface markers	Name	Category	Positive/negative
CD11b	*α*_b_ integrin	Adhesion molecule	Negative
CD104	*β*_4_ integrin	Adhesion molecule	Negative
CD14	Lipopolysaccharide	Receptor molecule	Negative
CD45	Leukocyte common antigen	Receptor molecule	Negative
CD79	MB-1	Receptor molecule	Negative
CD16	Fc receptor	Receptor molecule	Negative
HLA-DR	Human leukocyte antigen DR	Histocompatibility antigen	Negative
CD73	Ecto-5′-nucleotidase	Surface enzyme	Positive
CD13	Aminopeptidase	Surface enzyme	Positive
CD10	Endopeptidase	Surface enzyme	Positive
CD105	Endoglin	Adhesion molecule	Positive
CD49d	*α*_4_ integrin	Adhesion molecule	Positive
CD29	*β*_1_ integrin	Adhesion molecule	Positive
CD44	Hyaluronate	Receptor molecule	Positive
CD36	Thrombospondin	Receptor molecule	Positive
CD117	c-Kit	Receptor molecule	Positive
CD90	Thy-1	Extracellular matrix	Positive
CD146	Muc-18	Extracellular matrix	Positive
CD34	Hematopoietic progenitor cell antigen	Stem cell	Positive
CD133	Prominin-1	Stem cell	Positive

**Table 2:** Summary of *in vivo* application of ADSCs in experimental disease animals.

	Application	ADSC source	Administration route	Animal model	Results
Arrigoni et al. [58]	Bone	Rabbit	Surgical implantation	Rabbit	Bone formation with the ADSC was demonstrated by a significant increase in bone content
Chen et al. [48]	Bone	Human	Surgical implantation	Mice	Overexpression of miR-375 significantly enhanced ADSC osteogenesis both *in vitro* and *in vivo*
Li et al. [84]	Cartilage	Human	(i) Injection (ii) Surgical implantation	Rat	ADSCs showed a better inflammation-modulating property
				Rabbit	ADSCs with scaffold promoted cartilage regeneration in the long term
Cho et al. [49]	Cartilage	—	Surgical implantation	Rabbit	The quality of regenerative cartilage significantly improved in the ADSC group
Huurne et al. [101]	Cartilage	Mouse	Injection	Mice	The ADSC-based treatment could inhibit synovial thickening, the formation of enthesophytes associated with ligaments, and cartilage destruction
Yin et al. [107]	Cartilage	Rabbit	Surgical implantation	Rabbit	ADSCs containing the TGF immobilized scaffold better-promoted cartilage regeneration in defective articular cartilage
Hu et al. [113]	Nerve	Rat	Surgical implantation	Rat	Improved nerve regenerative ability for ADSC group compared to control
Kingham et al. [114]	Nerve	Human	Surgical implantation	Rat	Both ADSCs and stimulated-ADSCs could promote axon regeneration
Li et al. [116]	Nerve	Rat	Injection	Rat	ADSCs alleviated neurological deficits and reduced brain water content in rats
Durco et al. [132]	Nerve	Human	Surgical implantation	Mice	The number of nerve fibers and motor plates was higher in the ADSC group
Nagata et al. [134]	Myocardium	Mice	Transfusion	Mice	The transfusion of ADSCs exhibited the highest cardiac functional recovery and the high frequency of the recruitment to ischemic myocardium
Bobi et al. [140]	Myocardium	Pig	Injection	Pig	Myocardial perfusion at the anterior infarct border increased in ADSC-treated animals
Mori et al. [141]	Myocardium	Human	Surgical implantation	Porcine	Left ventricular remodeling attenuated and vascular density increased in the infarct border area in the ADSC group
Qiao et al. [145]	Myocardium	Rat	Injection	Rat	ADSC and dECM groups could increase angiogenesis, reduce the degree of fibrosis, and decrease infarct size
Ge et al. [151]	Liver	Pig	Injection	Pig	AST, ALT, T-BIL, and LDH were significantly decreased in ADSC treatment
Jiao et al. [152]	Liver	Pig	Injection	Pig	ADSC transplantation ameliorated the IRI-induced histopathological damage
Zhang et al. [153]	Liver	Pig	Injection	Pig	ADSC group promoted liver function recovery, reduced oxidative stress, and promoted liver regeneration
Yan et al. [154]	Liver	Canine	Injection	Canine	AST and ALT were rapidly decreased in ADSC treatment
Nishiwaki et al. [162]	Skin	Mice	Surgical implantation	Mice	ADSCs contributed to wound healing in a dorsal skin defect model in diabetic mice
Xiong et al. [165]	Skin	Human	Injection	Mice	ADSCs significantly accelerated the healing of skin wounds by promoting cell proliferation
Chou et al. [169]	Skin	Rat	Injection	Rat	The wound treated with ADM-ADSCs showed a significantly higher wound healing rate than other groups
Yu et al. [172]	Skin	Human	Surgical implantation	Mice	The neoskin formed in the presence of ADSC exhibited a thickness comparable to normal skin and possessed a highly organized collagen structure
Nakamura et al. [180]	Trachea	Rat	Surgical implantation	Rat	The mucociliary transport function was improved by ADSC transplantation
Jin et al. [181]	Bladder	Rat	Surgical implantation	Rat	The rat bladder repair effect was better in the ADSC group

**Table 3:** Summary of clinical studies on treatments with ADSCs.

	Study type	Application	Cell source	Administration route	Patients	Follow-up time (month)	Results
Lendeckel et al. [75]	Case	Bone	Human	Implantation	1	3	The CT scans showed new bone formation and near-complete calvarial continuity
Sándor et al. [76]	Case	Bone	Human	Implantation	13	12-52	Successful integration of the surrounding skeleton; the construct was noted in 10 of the 13 cases
Thesleff et al. [77]	Case	Bone	Human	Implantation	5	79.2	The clinical results are not superior to results achieved by conventional cranial repair methods
Vériter et al. [78]	Case	Bone	Human	Implantation	17	1-54	ADSC therapy is safe and feasible for clinical indications
Spasovski et al. [99]	Case	Cartilage	Human	Injection	9	18	MOCART score showed significant cartilage restoration
Song et al. [100]	Comparative	Cartilage	Human	Injection	18	24	The high-dose group of ADSCs exhibited the highest improvement
Pak et al. [102]	Case	Cartilage	Human	Injection	91	30	VAS improved 50–60% No major complications
Koh et al. [103]	Case	Cartilage	Human	Injection	44	24	94% patients excellent or good satisfaction; 76% abnormal repair
Bydon et al. [123]	Case	Nerve	Human	Injection	1	18	The subjective and objective measures showed different degrees of improvement
Konstanty-Kalandyk et al. [136]	Case	Myocardium	Human	Injection	15	1	No major complications
Houtgraaf et al. [148]	Comparative	Myocardium	Human	Injection	10	6	ADSC infusion could improve cardiac function and perfusion defects, accompanied by a 50% reduction in myocardial scar formation
Huang et al. [160]	Case	Liver	Human	Injection	6	6	The METAVIR score, Child-Pugh score, and MELD score showed positive results
Gotze et al. [161]	Case	Liver	Human	Injection	3	1-2	The reduction of liver stiffness and increase of ^13^C methacetin breath test outcome were observed
Jo et al. [176]	Case	Liver	Human	Injection	4	—	In these cases, they observed rapid coverage of the wound with the patient's regenerated tissue
